# A Modified Delphi Study for the Development of a Leadership Curriculum for Pediatric Oncology 

**DOI:** 10.31557/APJCP.2021.22.5.1659

**Published:** 2021-05

**Authors:** Mohammad Naghi, Marwa Rashad Salem

**Affiliations:** 1 *Faculty of Medicine, Helwan University, Egypt. *; 2 *Department of Public Health and Community Medicine, Faculty of Medicine, Cairo University, Cairo, Egypt. *

**Keywords:** Leadership, pediatric, modified Delphi, competencies, consensus

## Abstract

**Background:**

The broader construct of participatory system-wide leadership has not been highlighted in the pediatric oncology domain, although these skills are teachable. The researchers conducted the current study to develop a leadership competency curriculum for pediatric oncologists in Egypt.

**Methods:**

The study was carried out in three phases: (1) Conducting a literature review for the years 2013-2018 to identify the initial competencies of pediatric oncology leadership (2) Holding a meeting with a superior reference panel (SRP) to get a consensus on the initial list of competencies, and (3) Conducting a two-round modified Delphi survey.

**Results:**

Seventy-five invitees from 12 countries completed the first round (R1) of the survey. Of the 75 respondents to R1, 69 completed round 2 (R2) (92%). In R1, 73 competency items were surveyed for relevance to include in a pediatric oncology leader role curriculum. Thirty-seven items were judged for inclusion, while 36 items were judged for exclusion. In R2, the 37 competencies relevant for inclusion were presented in which participants were asked to check them as either IN or OUT. Finally, 17 competencies remained.

**Conclusion:**

The process resulted in 17 pediatric oncology leader role competencies. This final set of 17 competency items is considered as an important step towards reducing the variability in pediatric oncology education and practice that currently exists in Egypt. This is the initial step towards developing a learning and assessment toolkit for this imperative area of research and practice.

## Introduction

The sustainable development goals (SDGs) set a new agenda for health action at the global and national levels. This agenda calls for synergistic leadership across many levels including individuals, organizations, and systems (David Le Blanc, 2015). The commitment to deliver universal health coverage (UHC), a critical target of the health SDGs, is a challenge for health systems across the world. This calls for a new leadership model in health which can simultaneously propel change across health systems and the many social determinants of health (World Health Organization, 2013; United Nations General Assembly, 2015). 

In pediatric oncology, developing skills in leadership is mandatory during the transition of wide complex systems towards value-based care, quality improvement, safety, and efficiency taking into consideration that about 300,000 children aged 0 to 19 years old are diagnosed with cancer each year (Steliarova-Foucher et al., 2017). Most of these patients live in developing countries where quality care access is inadequate (Vineis and Wild, 2014), and only a few of these countries have established a strategy for cancer control (Brown et al., 2006). In addition lack of registries results in insufficient resource allocation and mobilization (Farmer et al., 2010). 

Pediatric oncology professionals need to implement efficient stewardship of resources and present unique personal skills and competencies (Uneke et al., 2012). Unfortunately, the broader construct of participatory system-wide leadership has not been highlighted in the pediatric oncology domain although these skills are teachable (Health Education and Training Institute [HETI], 2013). Therefore, the researchers conducted the current study to develop a leadership competency curriculum for pediatric oncologists in Egypt. This set of competencies can then serve as the groundwork for defining a full curriculum aiming to implant learning and assessment in developing the capacity within all pediatric oncology training programs.

## Materials and Methods

The study was carried out in three phases: (I) Literature review, (II) Meeting with a superior reference panel (SRP) to get a consensus on the initial list of competencies, and (III) a two-round modified Delphi survey. 

*Phase I: Literature review*

An initial framework of competencies focusing on the leader role competency items for the consensus meeting phase was identified from a review of published peer-reviewed literature including capabilities, competencies, skills, and/or behaviors in the area of health professional leadership .10-12 More references published in English between 2013 and 2019 on PubMed, HARVie, HOLLIS, Harvard Countway Library, EMBASE, Google search, and Google Scholar were accessed with the keywords: leadership, health care quality, health care management, competency, and pediatric oncology together with grey literature from a variety of oncology departments and leadership institutions.

The initial search generated more than 300 articles. Refining and limiting the search to the English language reduced this number to 110 articles. Excluding articles which do not address pediatrics or leadership and those which are not specific to pediatric oncology further reduced the articles’ number to 18. The principal investigator with research experience in pediatric oncology read these articles and further excluded those which are not related. Finally, 12 citations were included in the review. Citations and frameworks were reviewed for possible competencies and a list including 109 competencies was compiled (Supplement file 1 ). 

*Phase II: Consensus meeting with a superior reference panel (SRP)*

A consensus meeting was conducted with a superior reference panel (SRP) comprised of a Purposefully selected 8 pediatric oncology professors and associate professors from 6 different institutes as shown in Table 1. The purpose of this meeting was to discuss and perform brainstorming to bring together the set of initial leadership competency items before the first Delphi round. The questions guiding the discussion included: Why do you think we have to define a pediatric oncology leadership curriculum? How can we meaningfully integrate leadership in pediatric oncology practices? Would this curriculum be only applicable in Egypt, or it can have a global impact? What are your recommendations for the proposed pediatric oncology leadership curriculum?

For the consensus meeting session, there was one lead facilitator and one assistant moderator who was dedicated to audio-recording the group discussion session and taking written notes on a paper-based note-taking template. This group size was large enough to generate the needed dynamic in the discussion, yet small enough to remain manageable. The interviewers ended the interview when the conversation on the focus topics came to a natural end. 

The facilitator asked the participants to rate each of the 109 candidate competency items (revealed from the literature review phase) as per their importance for inclusion in a pediatric oncology leader role curriculum using the traffic light technique as follows: Green = good question, use it; Amber = not so good, needs modifying but still an important area; Red = not helpful, cannot be modified into anything useful or it should be discarded, , resulting in 73 items to be included in the Round I survey.

*Phase III: Modified Delphi survey*

A modified Delphi consensus technique was delivered through two rounds of online surveys to collect the experts’ agreement on various competencies. 

*Round I Delphi*

The initial invitation was sent to 380 pediatric oncologists from over 17 countries in which their contribution was requested in formulating the target curriculum through a Likert scaling question for all participants to measure their attitude towards specific items related to pediatric oncology practices. The principal investigator selected pediatric oncology physicians (seniors or oncology education experts) and a clinical pharmacist dealing with pediatric oncology cases to keep a homogenous sample of participants in order to anticipate stronger results with the Delphi method. All panelists had to meet the following criteria: experience and knowledge of pediatric oncology to ensure that they could contribute constructively to the process and willingness to participate in the Delphi discussions in English via e-mail. Considering the highly specific inclusion criteria and anticipated attrition at each round, there was no limit set to the number of participants and no sample size calculation was done. Sampling was purposive to ensure that those who were invited met the inclusion criteria. One reminder e-mail was sent to the non-responding invitees. Seventy five of the invitees agreed to contribute in the Delphi method. Overall, 75 complete responses to R1 were received (from 12 countries).

Invitations to participate were sent by e-mail and LinkedIn network. Seventy three items were included in the R1 survey which was delivered via the Survey Monkey online platform (www.surveymonkey.com) asking the participants to rate each of the 73 candidate competency items as per their importance for inclusion in a pediatric oncology leader role curriculum with a descriptive message clarifying the purpose of the research, ease of the survey platform’s use on both desktop and mobile devices, and the expected time to complete the survey (Supplement file 2). A scaling question was used and sent to all participants to measure their opinion towards specific items related to the pediatric oncology practices using a 5-point Likert scale from Very Important = 5, Important = 4, Moderately Important = 3, of Little Importance = 2, Unimportant = 1), resulting in 37 items to be included in the Round II survey.

*Round II Delphi outcomes*

The second (last) briefer survey was delivered via the Survey Monkey online platform. The R2 survey was sent to the 75 participants who responded to R1. Two reminder e-mails were sent via Survey Monkey TM platform to non-respondents and those who did not complete answering questions after two and three weeks from the first email of R2 survey Of the 75 invitees to R2, 69 invitees responded, representing a 92% response rate. 

In this round, the items for definite inclusion in the first round (37 items) were presented in which participants were asked to mark items as either IN (scored 1) or OUT (scored zero) (Supplement file 3). The experts were asked to mark each competency according to its relevance for inclusion in the framework. After the reviewers evaluated the items individually, the results were pooled to create an amended framework. A total of 17 competencies remained. 

*Statistical Analysis*

Data analysis was done simultaneously with data collection at each round of the Delphi discussions. Item analysis and descriptive statistics were performed on data received from experts who completed both Delphi rounds. Mean and standard deviations around each candidate item were computed. For R1, the consensus definition that was applied for ‘inclusion’ across each response was of a mean of ≥ 4 and SD ≤1 on the 5-point Likert scale (i.e. extremely or very important) (Hsu and Sandford, 2007). Items receiving a mean score of < 4 (regardless of SD) were designated items for ‘exclusion (Shaw et al., 2015). In R2, the consensus was defined as having been achieved if at least 75% of respondents scored the item as IN (scored for 1) as used by other investigators or greater agreement among experts (Sumsion, 1998). 

Qualitative data collected in the focus group discussion were analyzed using the thematic inductive analysis approach as described by Percy et al., (2015). 

Key patterns, meanings, and themes were identified and used in formulating the questionnaires for the first round discussions. Quotes highlighting unique and vivid experiences were identified during the analysis and have been presented in the results section.

*Ethical Considerations*

The Ethical Review Committee in the Faculty of Medicine at Helwan University revised and approved the study protocol. An electronically signed written Informed consent that were mailed was obtained from each participant after proper orientation of them regarding the study objectives. The researchers assigned a unique code to each expert and securely stored the data. Panelists remain anonymous to each other to allow freedom of expression without reservation. The study followed the principles of the Declaration of Helsinki

## Results

*Consensus Phase Results*

A consensus meeting was held with pediatric oncology staff for developing the initial framework. Their responses were analyzed, organized, and portrayed under the following broad lines:

*The importance of defining a pediatric oncology leadership curriculum*

All the inter-viewed physicians believed in the importance of defining a pediatric oncology leadership curriculum and how crucial it is to enhance the quality of the process of care for pediatric oncology patients. They all agreed that it is essential to fill the gaps between governance and management and define value-based care in pediatric oncology. 

*Integration of leadership in pediatric oncology practices*

The interviewed physicians agreed on some procedures for integration of leadership in pediatric oncology saying: “Analyzing the best practices in pediatric oncology quality and safety metrics, unifying systems, facilitating data migration which would take the research a leap forward in addition to training senior consultants, managers, and executives on leadership skills, tools, and competencies would visualize the big picture of oncology including all the external and internal stakeholders in people-centered care”.

*Generalizability of the proposed pediatric oncology leadership curriculum to low middle income countries (LMICs)*

All physicians agreed that Egypt has the advantage of belonging to low middle income countries (LMICs); this creates better opportunities in funding. Although the psychosocial support can differ from one country to another according to traditions and beliefs, the patient’s journey in oncology is still the same, so it can be modified in this specific part. All experts mentioned that all other parts of the curriculum can be used as the roadmap for pediatric oncology leaders in LMICs and that they have to be included as priorities in the leaders’ strategic plans as the competencies were specifically deficient in training programs , and are needed in practice.

*Recommendations for the proposed pediatric oncology leadership curriculum*

All the interviewed physicians recommended the following items to be integrated within the proposed pediatric oncology leadership curriculum:

Defining the interrelation with pharmaceutical companies, global healthcare leadership pros and cons, processes of legalizing a new treatment, annual protocols review and update, and the universal treatment regimens provided equally to all global similar diagnosis cases, so we can have realistic statistics for enhancing the accessibility of LMICs to the international societies to establish a pediatric oncology leadership society that unifies global children cancer treatment protocols. In addition to these items, they recommended more clarification on the strategy of early adoption of palliative treatment. 

All the interviewed physicians agreed that the conceptual framework should be short and has to be categorized. In addition, they agreed that analysis of results should be accurate and stick to transparency. Consequently, the 109 competency items were reduced into 73 items and re-organized according to the three main pillars of the global sustainable development goals Social (People), economic (Profit), and environmental (Planet) into the following 9 domains: Safety (7 items), Quality (13 items), Psycho-social (6 items), Education (7 items), Resource Allocation (11 items), Innovation (8 items), Evaluation & Monitoring (7 items), Resources Stewardship (5 items), and Leadership (9 items) (Supplement file 2). 

Descriptive statistics were sent to each panelist summarizing all the responses from R1 and their individual responses. The time lapse between the two Delphi rounds was one and a half month.

**Figure 1:**
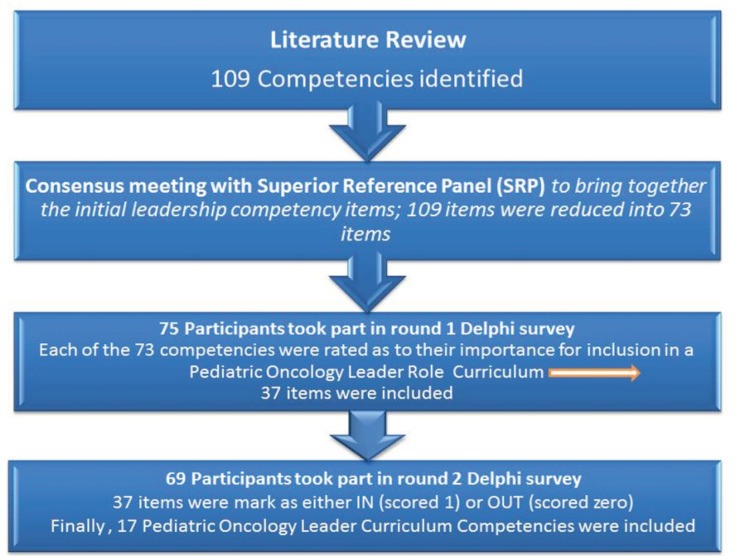
An Outline of the Process Defining theCurriculum Competency Profile for the Leader Role in Pediatric Oncology

An outline of the process defining the curriculum competency profile for the leader role in pediatric oncology is shown in Figure 1. This depicts the pathway through which the initial 73 candidate items evolved to reach the final set of 17 items. The resulting leader curriculum competency statements are shown in their final form in Table 3.

**Table 1:** Consensus Meeting Respondents’ Roles and Countries of Practice (n = 8)

Panelist	Qualifications	Institution Name	Roles and Responsibilities
1- M.S	MD. Pediatric Hematology/ Oncology	Mansoura University, Egypt	Professor/ Consultant POa
2- A.F	MD. Pediatric Hematology/ Oncology	Mansoura University	Professor/ Consultant PO
3- O. M	MD. MBA	Arab Academy for Science, Technology, and Maritime Transport	International Healthcare Consultant
4- Y.B	MD Pediatric Oncology	National Cancer Institute, Egypt/ Prince Sultan Military Medical City, Saudi	Associate Professor/Consultant PO/HSCT
5- A.D	MD. Pediatric Hematology/ Oncology	Mansoura University	Assistant Professor/Consultant PO
6- G. M	MD. Pediatric Hematology/ Oncology	Ain Shams University	Professor, Head of PO Department
7- A.S	MD. Pediatric Hematology/ Oncology	Alexandria University	Assistant Professor/Consultant PO
8- M.B	MD. Pediatric Hematology/ Oncology	Zagazig University	Professor, Head of PO Department

^a^PO, Pediatric oncology

**Table 2:** Round I Delphi Survey Respondents’ Role and Country of Practice (n = 75).

Response Number	Country	Institution Name	Specialty
25	1- Egypt	- Mansoura University - Alexandria University - Zagazig University - National Cancer Institute - Arab Academy for Science, Technology, and Maritime Transport	- Healthcare Leadership - Pediatric Oncology - Pediatric hematology - Pediatric Neuropsychology
3	2- Saudi	- King Faisal Specialist Hospital & Research Centre - King Fahad National Centre for Children's Cancer	- Pediatric Oncology
1	3- Qatar	- Hamad Medical Corporation	- Pediatric Oncology - Clinical Pharmacist
13	4- USA	- MD Anderson Cancer Center - St. Jude Children's Research Hospital - Yale University - University of Illinois College of Medicine - Memorial Sloan Kettering Cancer Center - UCSF Benioff Children's Hospital Oakland - Chicago's Public Research University - Children's Hospital Colorado - The Children's Mercy Hospital - Baylor College of Medicine	- Pediatric Oncology - Pediatric Surgical Oncology - Pediatrics - Neuro-Oncology - Pediatric Radiation Oncology
16	5- Canada	- Sickkids - McMaster University - Alberta Health Services	- Pediatric Hematology-oncology - Precision Medicine Consultant - Pediatric Oncology
4	6- China	- Shanghai Children’s Medical Center (SCMC) - West District of Union Hospital, Huazhong University of Science and Technology - Shanghai Jiaotong University School of Medicine - Peking Union Medical College - Wenzhou Medical University	- Pediatric Oncology - Pediatric Hematology-oncology - Pediatric Neuro-oncology
1	7- Kuwait	- Al Sabah NBK Pediatric Hospital	- Pediatric Oncology
2	8- Oman	- National Oncology Centre (NOC) Royal Hospital	- Pediatric Oncology - Pediatric Oncology Intensivist
2	9- Sweden	- UPPSALA UNIVERSITET	- Pediatric Oncology and Hematology - Pediatric Neuro-oncology
4	10- Germany	- klinikum-stuttgart - Heidelberg University Hospital	- Pediatric Oncology - Pediatric Neuro-oncology
1	11- Nigeria	- Usmanu Danfodiyo University Teaching Hospital (uduth), Sokoto	- Pediatric Oncology
3	12- India	- Cancer Institute, Chennai - Tata Memorial, Mumbai	- Pediatric Oncology - Pediatric Hemato Oncology
Total 75		- AIIMS, New Delhi	- Pediatric Nutrition –Oncology

**Table 3:** Final Pediatric Oncology Leader Curriculum Competencies

Rank	Curriculum	Agreed respondents of Total 69	%
1	Physician leaders involvement from the beginning in planning and implementation	65	0.94
2	Staff professional development through life-long learning	63	0.91
3	Drug availability	62	0.89
4	Early adoption strategy of palliative care	61	0.88
5	Maximization of outpatient care	60	0.86
6	International collaborative clinical trials	59	0.85
7	Transformation of cancer care from a high-cost to a high-value enterprise	58	0.84
8	Listening to what patients are saying	57	0.82
9	Implementation of innovative teaching tools, clinical guidelines,	56	0.81
10	Sufficient multidisciplinary staff, tumor boards	56	0.81
11	Encouragement of tele-health, remote care	55	0.79
12	Eradication of potential/actual medication errors	55	0.79
13	A formal program in an understandable language for patient/ family.	55	0.79
14	Protocol-adapted therapy	54	0.78
15	Hospital registry.	54	0.78
16	Infection control team	53	0.76
17	Inclusion of influential members of society,	52	0.75

## Discussion

This modified e-Delphi consensus process led to the determination of 17 leader role competencies for developing the pediatric oncologists’ leadership skills compared with the Global Mapping Project (Shaw et al., 2015). Which focuses on leveraging the organizational capacities. The 17 competencies defined through this process readily fit under the three core pillars of sustainable development goals . World Health Organization (2013) & United Nations General Assembly (2015). 

People: Eradication of medication errors, Infection control team, Maximizing outpatient care, Sufficient multidisciplinary staff/Tumor boards, Protocol-adapted therapy, Drug availability, Hospital registry, Listening to patients, Formal program in an understandable language for patient/family, Staff professional development through life-long learning, and Implementation of innovative teaching tools/clinical guidelines. Planet: Involvement of physicians from the beginning in planning and implementation, Encouraging remote care, and International collaborative clinical trials. Profit: Early adoption strategy of palliative care, Transformation of cancer care from a high-cost to a high-value enterprise, and Inclusion of influential members of society. These competencies are in keeping with the contemporary paradigms of team-based leadership in which they are essential to affect the change in healthcare environments.16 The importance of those leaders applying systems-based approaches is demonstrated in the quality improvement activities, (UNESCO, 2014). The 17 competencies are very critical especially with the current health system status, where service fragmentation, lack of awareness about the value-based care in pediatric oncology, and improper use of resources (both human and non-human resources) are dominant (Rodriguez-Galindo et al., 2015; Hsu and Sandford, 2007). 

The response rates and the consensus in the current study met expectations and are in harmony with the suggestion made by some authors (Rodriguez-Galindo et al., 2015). This might be explained by the inclusion of a heterogeneous group of pediatric oncology professionals of all types and at all levels, including those in training as key members of pediatric oncology teams providing patient care, teaching, and undertaking service improvements who clearly have an important perspective on the essential competencies to be included in the proposed leadership curriculum. Additionally, the entire study was completed through both electronic communication and face-to-face meetings that contributed to the high consensus and response rate.

The basic objective of this study is to develop a leadership competency curriculum for pediatric oncologists.This is in accordance with a previous study conducted to develop a curriculum for pediatric emergency residents (Mitzman et al., 2017). 

The current study did not address how to teach these competencies as this will vary widely based on patient populations, resources, experts availability, and institutional practice. Future work should be performed to develop the best practices for delivering these competencies and developing assessments for measuring the competencies’ achievements.

This modified Delphi consensus process resulted in the definition of a set of 17 competency items for leader role in pediatric oncology. Although the final recommendations did not reach complete consensus, the final set of competencies is considered as an important step towards reducing the variability in pediatric oncology education and practice that currently exists in Egypt. The next step of developing learning and in-training assessment methods by which to ensure attainment of these competencies is already well underway.

## Author Contribution Statement

Mohammad Naghi contributed in managing the literature searches, the practical part, and writing. Marwa Rashad Salem contributed in data management and writing. All authors shared in writing, drafting and approving the final manuscript and all approve equal share in the study.
